# Commentary: Duty to Warn: Antidepressant Black Box Suicidality Warning is Empirically Justified

**DOI:** 10.3389/fpsyt.2020.00363

**Published:** 2020-04-30

**Authors:** Wayne K. Goodman, Eric A. Storch

**Affiliations:** Menninger Department of Psychiatry and Behavioral Sciences, Baylor College of Medicine, Houston, TX, United States

**Keywords:** suicidality, Black Box, activation syndrome, behavioral toxicity, antidepressants

In a recent issue of Frontiers in Psychiatry, Spielmans et al. (1) defend the 2004 decision of the Food and Drug Administration (FDA) to issue a Black Box warning on the potential risk of suicidality in youth being administered antidepressant medications. Some researchers have claimed this action had a chilling effect on prescribing and led to an increase in youth suicides (2). Spielmans et al. (1) are to be commended for carefully reviewing and critiquing the evidence behind such claims of unintended consequences and for offering an alternative interpretation that the Black Box warning was and remains empirically justified. They are equally careful to point out the limitations of the available data, which are mostly correlational (not causal), that led to their own conclusions.

As Chair of the FDA Psychopharmacological Drugs Advisory Committee proceedings that led to the Black Box warning, one author of this commentary (WKG) had a front row seat at hearings in which both data and public testimonials from a variety of viewpoints were presented. In addition to the 2004 meeting that focused on pediatric data, another meeting was convened in 2006 in which the FDA presented data compiled from nearly 100,000 subjects across the lifespan who participated in 372 randomized controlled trials comparing antidepressant medications to placebo across a number of indications (3). The relative risk of suicidality in the drug versus placebo groups showed an interesting pattern across the age range (see **Figure 1**). As noted in 2004, the odds for suicidality were higher for active drug relative to placebo for individuals under age 25 years. However, for older individuals, active drug was actually protective against suicidality (both ideation and behavior) compared to placebo and this effect was most pronounced for the oldest cohorts [see Forest plot in Stone et al. (3)].

**Figure 1:**
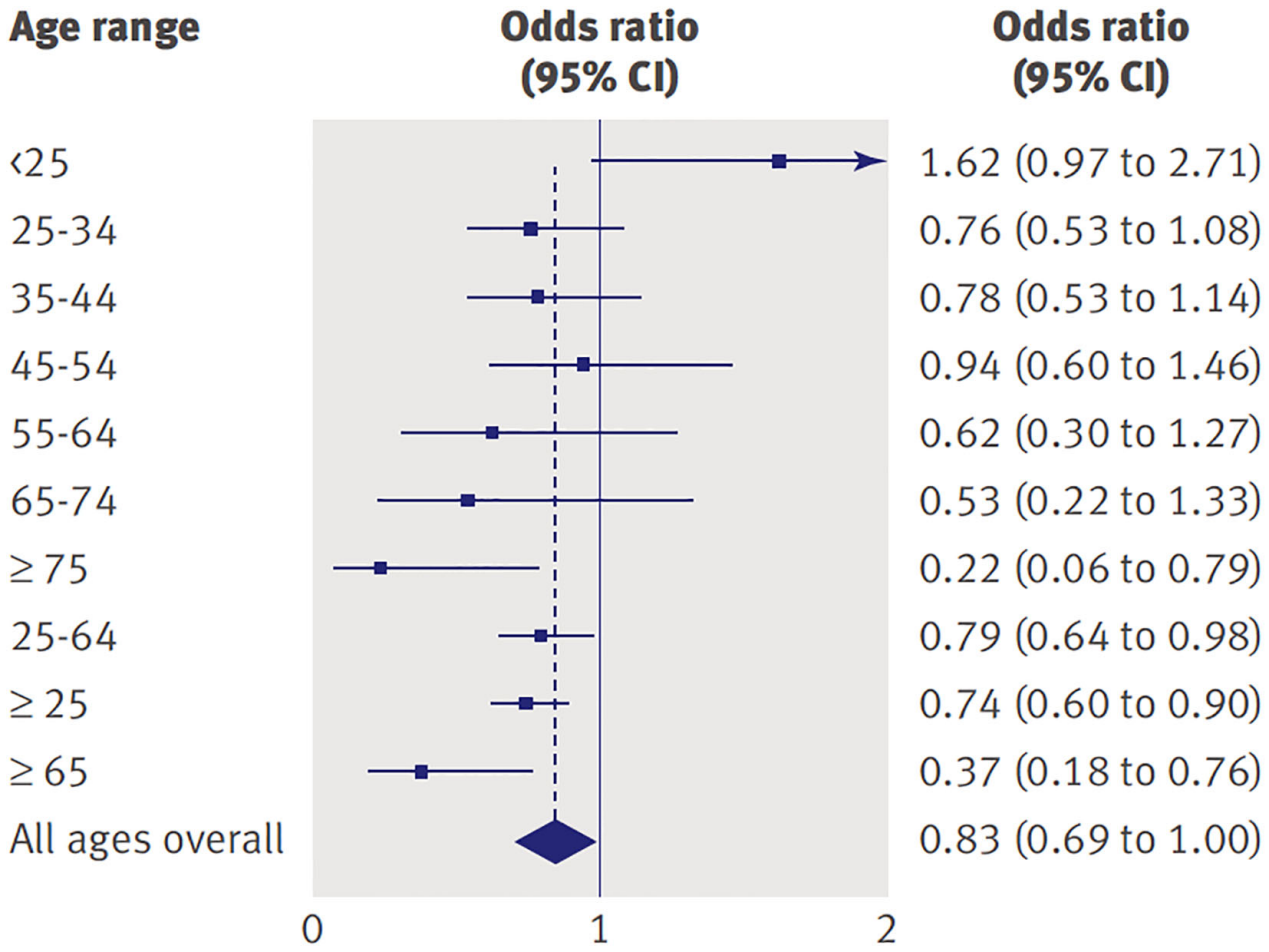
Odds of suicidality (ideation or worse) for active drug relative to placebo by age in adults with psychiatric disorders. Reprinted with permission from (3).

What might account for this relationship between age and risk of suicidality associated with antidepressants? Although the literature is inconclusive (4), susceptibility to and nature of SSRI-induced behavioral side effects may be a function of brain maturation occurring throughout childhood and adolescence, ending during early adulthood. This hypothesis has been supported by various rodent models (5), as well as limited human studies (6). For example, although not direct support of the link between brain maturation and SSRI-induced activation, evidence of increased dorsolateral prefrontal cortex grey-matter volume among depressed youth relative to healthy controls may suggest differing brain maturational processes (7, 8) for adolescents with psychiatric disorders. As various neural correlates (e.g., amygdala connectivity) have predicted attenuated antidepressant treatment response (9, 10), it is possible that such developmental alterations in brain maturation also relate to increased possibility of SSRI-induced behavioral side effects. Therefore, it is not surprising that the effects of antidepressants in the developing brain, both in regard to efficacy and side effects, might be different compared to adult brains. In 2007, Goodman et al. (11) hypothesized that antidepressants might induce “behavioral toxicity” in some susceptible youth that could act as a precursor for suicidality if undetected. Those preceding behavioral effects can assume various manifestations including activation syndrome (11). Once antidepressant induced suicidality is conceptualized as a behavioral side effect, it becomes less surprising that it can occur at higher rates in the active versus placebo arms of a study.

How to best monitor for early signs of adverse behavioral effects during treatment that could evolve into suicidality remains an unanswered question. The Treatment-Emergent Activation and Suicidality Assessment Profile (TE-ASAP) was developed to assist parents and child and adolescent psychiatrists with monitoring behavioral side effects during antidepressant treatment of youth (12). This 38-item scale covers several constructs often associated with activation syndrome, including irritability, hyperactivity, disinhibition, motor restlessness, externalizing behaviors and hypomanic symptoms. In a study of 56 youth with obsessive compulsive disorder (OCD) who participated in a double-blind trial of sertraline versus placebo, the TE-ASAP showed acceptable psychometric properties (12). The study was too small to assess how well it identified participants at risk of suicidality.

It seems inconceivable that antidepressants would induce suicidality in the absence of other associated or antecedent behavioral changes. The essential message of the Black Box is to remind prescribers and consumers about the importance of monitoring closely for adverse behavioral changes during the initiation of (or changes in) antidepressant therapy in order to reduce the risk of suicidality in patients through age 24 years. The intention was not to discourage appropriate prescribing of antidepressants for youth with depression, OCD or anxiety disorders. In fact, some evidence, as reviewed by Fornaro et al. (13), suggested substantial reductions in antidepressant medication prescriptions in children and adolescents following the Black Box Warning (14). In addition, the period following the Black Box Warning was associated with an increased number of attempted/completed suicides (15), increased utilization of antipsychotic and benzodiazepine medications (2) and no significant change in use of psychotherapy (16). For the majority of these patients, the benefits of antidepressants greatly outweigh the risks. Nevertheless, we agree with Spielmans et al. (1) that prescribers have a “duty to warn” and highlight the need for adequate training for all potential prescribers during medical school and residency programs.

## Author Contributions

Both authors made substantial contributions to conceptualization and preparation of this manuscript.

## Conflict of Interest

The authors declare that the research was conducted in the absence of any commercial or financial relationships that could be construed as a potential conflict of interest.
